# Eosinophils Are More Strongly Relevant to Allergic Sensitization Than Basophils in Pediatric Adenotonsillar Hypertrophy

**DOI:** 10.3389/fped.2021.598063

**Published:** 2021-03-31

**Authors:** Juanjuan Zou, Yan Yang, Qiang Fu, Huayang Liu, Chao Zhang, Lili Liu, Yan Wang, Yanzhong Li

**Affiliations:** ^1^Department of Otorhinolaryngology, Qilu Hospital of Shandong University, NHC Key Laboratory of Otorhinolaryngology (Shandong University), Jinan, China; ^2^Department of Pediatrics, Qilu Hospital of Shandong University, Jinan, China

**Keywords:** eosinophil, basophil, allergen, adenoid and tonsil, Immunoglobulin E

## Abstract

The relationship between eosinophils/basophils and allergic sensitization is not clear in pediatric adenotonsillar hypertrophy (ATH). The objective of this study is to investigate the relationship between eosinophil/basophil counts and peripheral specific IgE levels, and identify the common allergens in children with ATH. We initially screened 1,031 consecutive children who underwent adenotonsillectomy in our department from June 2018 to June 2019, and finally included 676 children. The eosinophil count, basophil count, and levels of specific IgE were collected. Correlations between two quantitative variables were assessed using the Pearson or Spearman coefficient. Logistic regression analyses were performed to evaluate the odds ratios (ORs) for atopy after controlling for age, sex, vitamin D, BMI, and visiting season. Both the eosinophil and basophil counts in atopic participants were significantly higher compared to non-atopic participants. The eosinophil count correlated with the levels of IgE specific to all allergens, and eosinophilia was independently associated with all tested atopy allergens other than atopy to dander after multivariate adjustment. Additionally, the basophil count correlated with the IgE levels specific to A. *alternate* and food mix, and basophilia was still significantly associated with atopy to food mix after multivariable adjustment. Furthermore, among allergic participants, D. *farinae* was the most prevalent allergen, followed by food mix, D. *pteronyssinus*, and A. *alternata*. In conclusion, eosinophils were more relevant to allergic sensitization than basophils, with eosinophils being significantly associated with all tested atopy allergens apart from dander, and basophils being associated with atopy to food mix. Furthermore, D. *farinae* was the most prevalent allergen and may be indicative of desensitization therapy.

## Introduction

As a major part of the Waldeyer's ring, adenoids and tonsils play an important role in immune defense. However, this function may be disturbed when they enlarge to cause narrowing of the airway due to chronic stimulation. Adenotonsillar hypertrophy (ATH) is a common disorder in children affecting ~34% of the general pediatric population and 42–70% of the children visiting otolaryngologists (1). It is the most common reason for upper airway obstruction in children and can lead to a series of sequelae such as mouth breathing, obstructive sleep apnea, otitis, sinusitis, and craniofacial anomalies (2). Although the reasons for ATH remain unelucidated, some studies have suggested allergies as a risk factor. Sadeghi-Shabestari et al. (3) found that 70.3% of ATH children tested positive in a skin prick test, while only 10% of the non-ATH children tested positive, and children hypersensitive to dust mites were more susceptible to ATH (4). In addition, Papatziamos et al. (5) demonstrated that IgE-positive plasma cells were found in the adenoids of atopic children, while non-atopic adenoids contained none or very few IgE-positive cells. All these reports indicate that there is a close relationship between allergies and ATH.

An allergic reaction includes an early and late phase, and the late phase may lead to clinical manifestations due to the infiltration of various inflammatory cells including eosinophils, which are one of the two key effector cells in allergic inflammation (6). Although eosinophils can cause bronchial hyperreactivity and respiratory inflammation, the specific role and importance of eosinophils in allergic respiratory responses is not clear (7, 8) and there are few studies that have investigated the relationship between eosinophil count and allergies in ATH children. Only one study has demonstrated that the eosinophil count in adenotonsillar tissue was significantly higher in sensitized children, and the clinicians could accordingly distinguish the allergic patients (9). However, the study sample was small, confounding factors were not adjusted, and more importantly, this approach is not practical for ATH children who do not need surgery. Although basophils are the least abundant peripheral blood leukocytes, comprising <1.0%, they play an important role in the immune response (10, 11). Nevertheless, few studies have addressed the role of basophils in ATH children. Considering that serum specific IgE (sIgE) measurement is the most commonly used *in vitro* diagnostic approach for allergies, and easy to perform in young children (12), it is widely used in children.

Therefore, we hypothesize that there may be an independent relationship between eosinophil/basophil counts and allergies in ATH children. The aim of this large cross-sectional study performed in ATH children was to (1) investigate the relationship between the peripheral specific IgE level and blood eosinophil/basophil counts, respectively and (2) clarify the common allergens in ATH children.

## Methods

### Study Population

**Figure 1:**
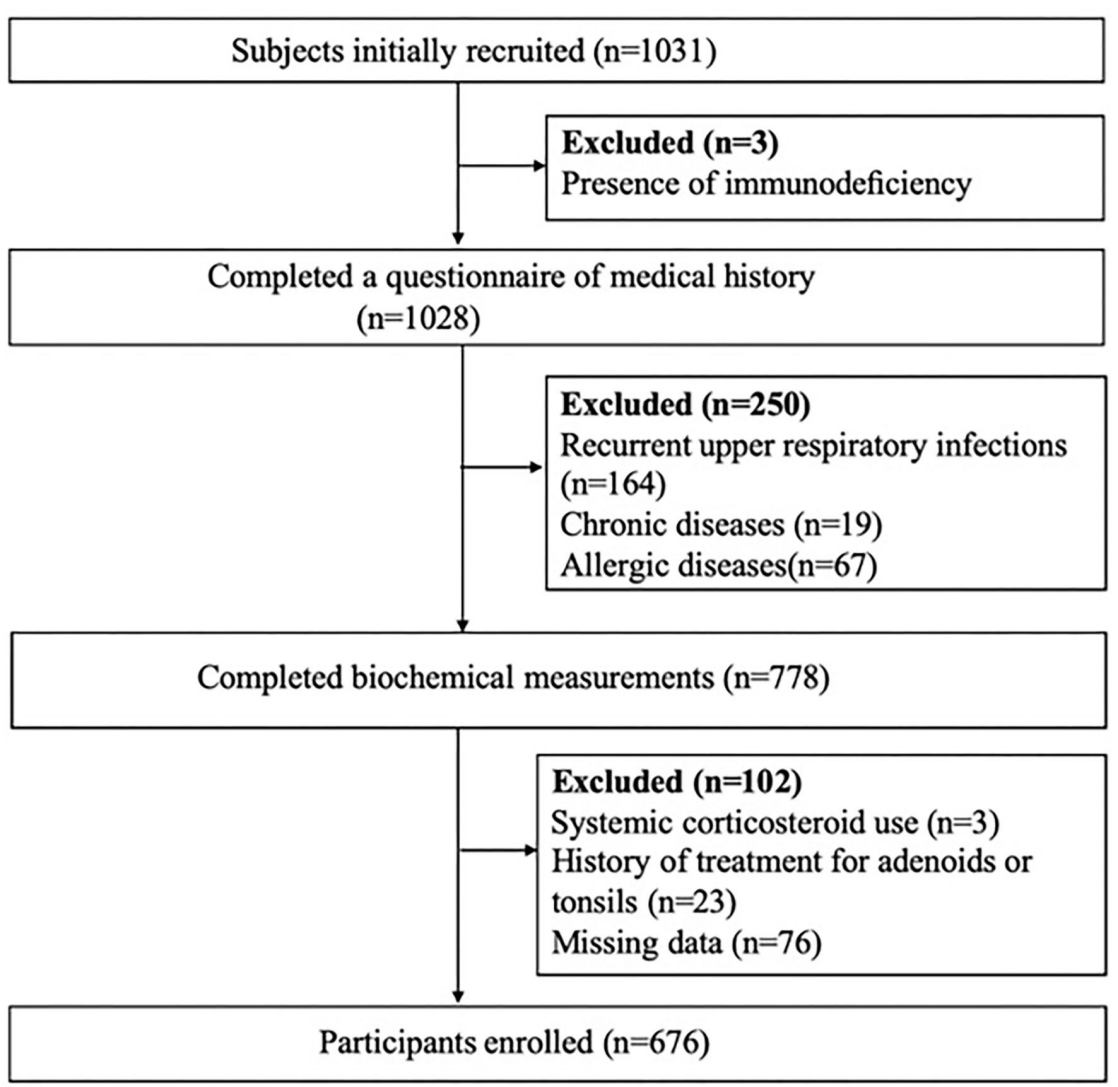
Flow chart of study population enrollment.

Initially, 1,031 consecutive children who underwent adenotonsillectomy at the department of otorhinolaryngology, Qilu Hospital of Shandong University between June 2018 and June 2019 were retrospectively recruited. The inclusion criteria included: (1) a diagnosis of obstructive sleep apnea, (2) failed medical treatment for ATH during the previous 3 months, or (3) ≥ grade 2 enlargement of tonsils plus ≥ grade 3 enlargement of adenoids or an adenoid nasopharyngeal ratio ≥ 0.62 on a nasopharyngeal lateral radiograph. The exclusion criteria were as follows: (1) presence of immunodeficiency; (2) recurrent upper respiratory infections; (3) chronic diseases, such as chronic kidney disease, psychiatric disorder, or hyperparathyroidism; (4) allergic diseases, such as allergic rhinitis, asthma, and atopic dermatitis; (5) systemic corticosteroid use in the previous 30 days; (6) history of treatment for adenoids or tonsils; and (7) missing clinical data. Ultimately, a total of 676 children with ATH were included (Figure 1).

Tonsillar size was graded as follows: grade 1: the tonsils are in the tonsillar pillar; grade 2: the tonsils protrude out of the tonsillar pillar; grade 3: the tonsils reach the midpoint between the anterior tonsillar pillar and uvula; and grade 4: the tonsils reach the uvula (13). Adenoidal size was graded based on the percentage of adenoid tissue occupying the rhinopharyngeal cavity: grade 1 (<25%); grade 2 (<50%); grade 3 (<75%); and grade 4 (>75%). ATH was assessed by two otolaryngologists (YW and YL), and any discrepancies were addressed by negotiation with the third reviewer (QF).

All participants provided written informed consent. This study was approved by the Internal Review Board of the Institutional Ethics Committee of Qilu Hospital of Shandong University (KYLL-2020(KS)-228) and was conducted in accordance with the tenets of the Declaration of Helsinki.

### Measurements

Fasting blood samples were collected from all participants and analyzed in the hospital laboratory using standard procedures. Eosinophilia was defined as eosinophil count >0.5 × 10^9^ cells/L (14) and basophilia was defined as basophil count >0.06 × 10^9^ cells/L according to the standards of the hospital laboratory. Height was measured with a meter ruler, weight was measured with a weighing scale using standard procedures, and body mass index (BMI) was calculated as weight (kg)/height^2^ (m^2^).

### Assessment of Serum IgE

The levels of IgE specific to *D. pteronyssinus, D. farinae, Penicillium notatum, Cladosporium herbarum, Aspergillus fumigatus*, dog dander, cat epithelia, egg, milk, wheat flour, fish, peanut, soybean, common ragweed, mugwort sagebrush, willow, and poplar were measured with the ImmunoCAP 100 system (Uppsala, Sweden). An sIgE value ≥0.35 kU/L was considered positive and atopy was defined as being positive to at least one allergen (12). The sIgE values were graded as follows: <0.35 kU/l, 0.35–0.69, 0.70–3.49, 3.50–17.4, 17.5–49.9, 50.0–99.9, ≥100 kU/L. Total allergen sIgE score was calculated as the sum of all the sIgE grades.

### Statistical Analysis

All statistical analyses were performed with the SPSS 24.0 software (SPSS, Inc.; Chicago, IL, USA). The participants were divided according to the eosinophil count into the non-eosinophilia group and eosinophilia group. Data were presented as mean ± standard deviation (SD) for continuous variables and percentage for categorical variables. Differences between the two groups were examined using the independent samples *t*-test or Mann-Whitney *U* for normally distributed or skewed variables, respectively. Categorical variables were compared using the chi-square test. Correlations were estimated using Pearson's correlation test for normally distributed data and Spearman's correlation test for non-normally distributed data. Binary logistic regression analysis was performed to assess the relationship between eosinophilia and sIgE after adjusting for age, sex, vitamin D, BMI, and visiting season. Furthermore, in order to fully evaluate the associations of sIgE with eosinophil and basophil, we performed the aforementioned analysis with the percentage of eosinophils and basophils. A two-tailed *P*-value <0.05 was considered to be significant.

## Results

### Basic Characteristics of the Study Participants

**Figure 2:**
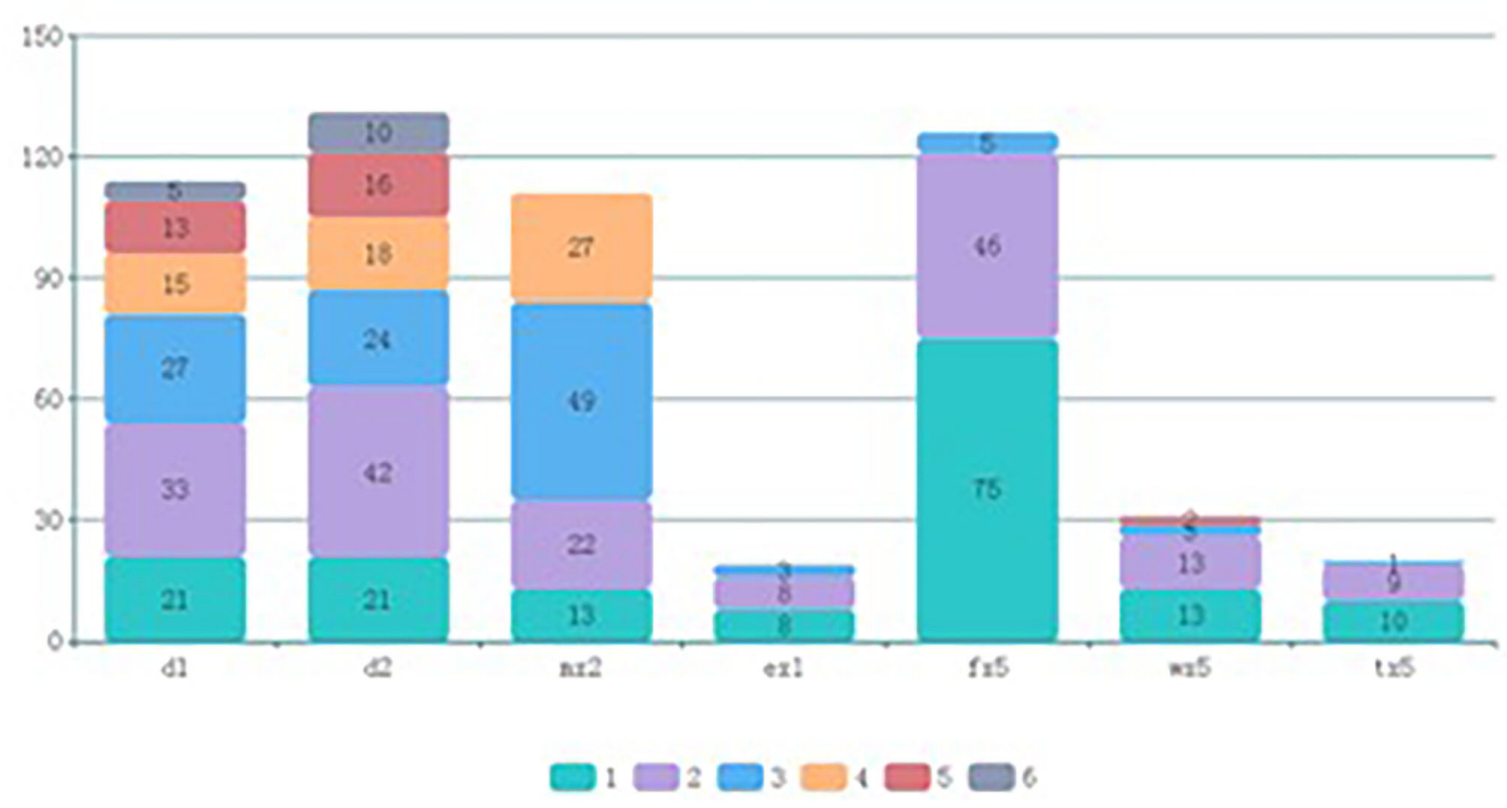
Types and grades of allergens in positive participants. d1, *D. pteronyssinus;* d2, *D. farina;* mx2, *A. alternata;* ex1, dander; fx5, food mix; wx5, mugwort; tx5, willow.

The flow chat is shown in Figure 1. The 676 participants were divided according to eosinophil count into the non-eosinophilia group (*n* = 624) and eosinophilia group (*n* = 52). The basic characteristics are shown in Table 1. The children with eosinophilia exhibited higher total allergen sIgE scores than children without eosinophilia (5.71 ± 5.60 vs. 1.65 ± 3.04, *P* < 0.001), and the percentage of children who were positive for serum sIgE to at least one of the examined allergens was higher in the eosinophilia group compared to the non-eosinophilia group (82.7 vs. 38.9%, *P* < 0.001). The prevalence of sensitivity to *D. pteronyssinus, D. farinae, A. alternate*, food mix, mugwort, and willow was significantly higher in the eosinophilia group (all *P* < 0.05), while there was no significant difference between the two groups with regard to sensitivity to dander. In addition, among the eosinophilia children, 76.9% were sensitized to more than two allergens. The types and grades of allergens in positive participants are shown in Figure 2.

**Table 1:** Basic characteristics of the study subjects.

	**Non-eosinophilia (*N* = 624)**	**Eosinophilia(*N* = 52)**	***P*-value**
**Characteristics**			
Age (years)	6.00 ± 2.87	5.65 ± 2.27	0.826
Male, *n* (%)	425, 68.1	44, 84.6	0.013
Vitamin D, (ng/mL)	25.42 ± 10.13	27.61 ± 8.25	0.025
Basophil count (*10^9^/L)	0.04 ± 0.02	0.06 ± 0.03	<0.001
Body mass index (kg/m^2^)	17.49 ± 4.33	16.61 ± 4.44	0.176
**Season**			
Spring	178, 28.5	10, 19.2	0.028
Summer	240, 38.5	31, 59.6	
Autumn	111, 17.8	5, 9.6	
Winter	93, 14.9	6, 11.5	
**Atopy**			
Total allergen sIgE score	1.65 ± 3.04	5.71 ± 5.60	<0.001
Atopy, *n* (%)	243, 38.9	43, 82.7	<0.001
Polysensitization, *n* (%)	169, 27.1	40, 76.9	<0.001
Sensitivity to *D. pteronyssinus, n* (%)	95, 15.2	19, 36.5	<0.001
Sensitivity to *D. farinae, n* (%)	110, 17.6	21, 40.4	<0.001
Sensitivity to *A. alternata, n* (%)	86, 13.8	25, 48.1	<0.001
Sensitivity to dander, *n* (%)	16, 2.6	3, 5.8	0.179
Sensitivity to food mix, *n* (%)	107, 17.1	19, 36.5	0.001
Sensitivity to mugwort, *n* (%)	24, 3.8	7, 13.2	0.001
Sensitivity to willow, *n* (%)	14, 2.2	6, 11.5	<0.001

*Data are presented as mean ± SD or percentage*.

### Association of sIgE Level With Eosinophil Count and Basophil Count, Respectively

**Figure 3:**
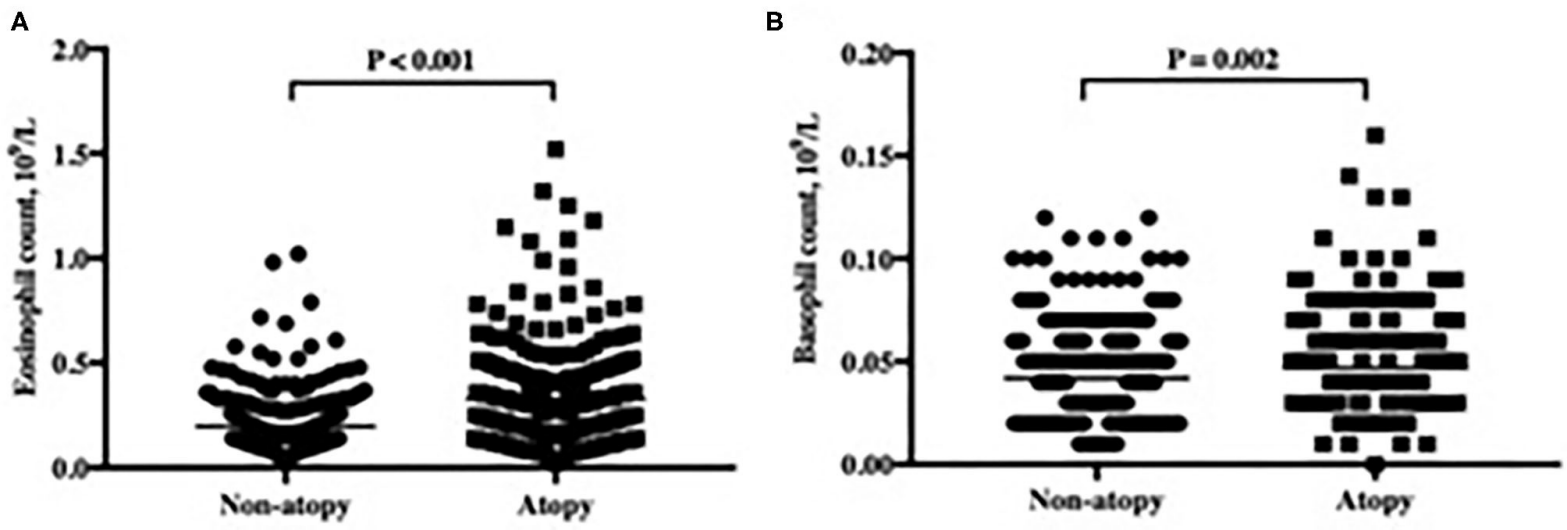
The counts of eosinophil **(A)** and basophil **(B)** in atopic and non-atopic subjects.

As shown in Table 2, the levels of IgE specific to *D. pteronyssinus, D. farinae, A. alternate*, dander, food mix, mugwort, and willow, as well as the total allergen sIgE score were significantly associated with the eosinophil count. The correlation coefficient was 0.386 for the total allergen sIgE score, and the best correlation among sIgE was for *A. alternate* (*r* = 0.331, *p* < 0.001). Furthermore, the eosinophil count was significantly higher in atopic children than in non-atopic children (*p* < 0.001) (Figure 3A).

**Table 2:** Correlations between levels of sIgE and eosinophil count, basophil count, respectively.

	**Eosinophil count**		**Basophil count**	
	***r***	***P***	***r***	***P***
*D. pteronyssinus*	0.251	<0.001	0.050	0.195
*D. farinae*	0.233	<0.001	0.021	0.589
*A. alternata*	0.331	<0.001	0.118	0.002
Dander	0.227	<0.001	0.041	0.284
Food mix	0.205	<0.001	0.100	0.009
Mugwort	0.198	<0.001	0.045	0.239
Willow	0.228	<0.001	−0.002	0.960
Total allergen sIgE score	0.386	<0.001	0.126	0.001

With regard to the basophil count, it was only significantly associated with the levels of IgE specific to *A. alternate* and food mix, as well as the total allergen sIgE score. Moreover, the basophil count in atopic children was significantly higher than in non-atopic children (*p* = 0.002) (Figure 3B).

In order to fully evaluate the associations of sIgE with eosinophils and basophils, we performed the aforementioned analysis with the percentage of eosinophils and basophils (Supplementary Materials). The results showed that the total allergen sIgE score and levels of IgE specific to all tested allergens were significantly associated with the percentage of eosinophils, while the percentage of basophils was only significantly associated with the total allergen sIgE score (Supplementary Table 1).

### Association of sIgE Level With Eosinophilia and Basophilia, Respectively

Based on the correlation analysis, we employed logistic regression analyses with multivariate adjustment to identify the association of atopy with eosinophilia and basophilia, respectively. As shown in Table 3, eosinophilia was associated with atopy to all tested allergens except atopy to dander, and the association remained significant after adjusting for multiple variables including age, sex, vitamin D, and season. The odds ratios (ORs) and 95% confidence intervals (95% CI) for atopy to *D. pteronyssinus, D. farinae, A. alternata*, food mix, mugwort, and willow were 3.431 (1.835, 6.413), 3.483 (1.885, 6.437), 5.634 (3.073, 10.328), 3.184 (1.696, 5.980), 3.402 (1.335, 8.670), and 7.089 (2.442, 20.574), respectively. In the case of basophilia, after multivariable adjustment, it was still significantly associated with atopy to food mix with ORs (95% CI) of 2.080 (1.238, 3.496). Besides, it was also significantly associated with atopy to mugwort and willow with ORs (95% CI) of 2.654 (1.150, 6.124) and 3.431 (1.240, 9.489), respectively (Table 4).

**Table 3:** Associations of eosinophilia with atopy.

	**Unadjusted**		Multivariable adjusted^*^	
	**OR**	**95% CI**	**OR**	**95% CI**
*D. pteronyssinus*	3.206	(1.750, 5.872)	3.431	(1.835, 6.413)
*D. farinae*	3.165	(1.753, 5.716)	3.483	(1.885, 6.437)
*A. alternata*	5.792	(3.212, 10.446)	5.634	(3.073, 10.328)
Dander	2.186	(0.619, 7.718)	1.968	(0.536, 7.229)
Food mix	2.782	(1.524, 5.077)	3.184	(1.696, 5.980)
Mugwort	3.889	(1.589, 9.515)	3.402	(1.335, 8.670)
Willow	5.683	(2.086, 15.483)	7.089	(2.442, 20.574)
Atopy	7.491	(3.588, 15.642)	8.424	(3.869, 18.345)
Polysensitization	8.974	(4.598, 17.517)	9.408	(4.680, 18.911)

* *Adjusted for age, sex, vitamin D, BMI and visiting season*.

**Table 4:** Associations of basophilia with atopy.

	**Unadjusted**		Multivariable adjusted^*^	
	**OR**	**95% CI**	**OR**	**95% CI**
*D. pteronyssinus*	0.709	(0.373, 1.349)	0.757	(0.394, 1.457)
*D. farinae*	0.786	(0.436, 1.417)	0.863	(0.472, 1.576)
*A. alternata*	1.623	(0.950, 2.775)	1.546	(0.896, 2.669)
Dander	2.340	(0.822, 6.658)	2.212	(0.758, 6.454)
Food mix	2.035	(1.237, 3.349)	2.080	(1.238, 3.496)
Mugwort	2.770	(1.233, 6.221)	2.654	(1.150, 6.124)
Willow	2.841	(1.063, 7.590)	3.431	(1.240, 9.489)
Atopy	1.295	(0.834, 2.013)	1.339	(0.853, 2.103)
Polysensitization	1.527	(0.969, 2.407)	1.523	(0.957, 2.426)

* *Adjusted for age, sex, vitamin D, BMI and visiting season*.

In terms of the percentage of eosinophils and basophils, after multivariable adjustment, the percentage of eosinophils was associated with atopy to all tested allergens except atopy to dander and food mix, while the percentage of basophils was not associated with atopy to any allergen (Supplementary Tables 2, 3).

## Discussion

Our study results indicate that both eosinophils and basophils were independently associated with allergic sensitization in ATH children, but eosinophils were more relevant than basophils after adjusting for multiple variables. In particular, eosinophilia was independently associated with atopy to *D. pteronyssinus, D. farinae, A. alternata*, food mix, mugwort, willow, and polysensitization. In addition, among positive participants, *D. farinae* was the most prevalent allergen, followed by food mix, *D. pteronyssinus*, and *A. alternata*.

Numerous previous studies have demonstrated a relationship between allergies and ATH, even suggesting allergies as a risk factor for ATH (3, 5). Although eosinophils and basophils are both important cells involved in allergic reactions, few studies have investigated the relationship between atopy and eosinophils/basophils in ATH children. Therefore, we performed this large sample size study in order to explore the relationship between blood eosinophil count and sIgE. We found that the eosinophil count was significantly higher in atopic participants, and the levels of IgE specific to *D. pteronyssinus, D. farinae, A. alternate*, dander, food mix, mugwort, and willow were related to eosinophil count. In addition, after controlling for confounding factors including age, sex, vitamin D, BMI, and visiting season, the independent associations between eosinophilia and atopy to the above allergens persisted except for atopy to dander. This indicates that eosinophilia may be utilized to estimate the risk of atopy in ATH children.

Basophils are the prime early producers of cytokines which are crucial for allergic responses, but they are often underestimated because they are less numerous than other types of leucocytes (10, 11). To the best of our knowledge, this is the first study to evaluate the relationship between basophil count and multiple sIgE production, especially in ATH children. In this study, we found that the basophil count in atopic ATH children was significantly higher, and in particular, it was related to the levels of sIgE to *A. alternata* and food mix. Furthermore, after multivariate adjustment including for vitamin D, which has been associated with milk allergy (15), basophilia was still associated with atopy to food mix. The underlying mechanism may be attributed to the fact that basophils can endogenously produce interleukin 4 and subsequently promote the development of IgE-mediated ovalbumin allergy (16). Interestingly, basophilia was also significantly associated with mugwort and willow atopy, but the association with *A. alternata* atopy disappeared after multivariate adjustment. The reason for this inconsistency may be interactions among the adjusted confounding factors.

As to allergens, we found that, among positive ATH children, *D. farinae* was the most prevalent allergen, followed by food mix, *D. pteronyssinus*, and *A. alternata*. So far, although a specific allergen in ATH children was not identified, the most common allergens were among the above allergens. For instance, Zhang et al. (13) concluded that milk was the most prevalent allergen in ATH children, followed by *D. pteronyssinus and D. farina*; Muhammed et al. (17) found that the most common allergen among adenoid hypertrophy children was *D. pteronyssinus*; and Mahmut et al. (18) revealed that sensitivity to *A. alternata* was more frequent in allergic rhinitis patients with adenoid hypertrophy than those without adenoid hypertrophy. These findings indicate that tonsillectomy and adenoidectomy may be beneficial in children with atopy to *D. farina*, food mix, *D. pteronyssinus*, or *A. alternata*, and large-scale epidemiological investigations are urgently needed.

To the best of our knowledge, this is also the first study to compare the eosinophil count with the basophil count for an association with allergic sensitization in ATH children and reveals the important role of eosinophils and basophils in allergic in?ammation among ATH children. However, the study has some limitations. First, this cross-sectional study could not infer causality because of lack of temporality. Second, total IgE and some allergens such as seafood, pollens, and fruits were not tested. Finally, although we adjusted for confounding factors, other variables, such as obesity, were not accounted for.

In conclusion, our study evaluated the relationship between eosinophils/ basophils and atopy in ATH children, and found that eosinophils are more relevant than basophils to allergic sensitization, with eosinophils being significantly associated with atopy to *D. pteronyssinus, D. farinae, A. alternata*, food mix, mugwort, and willow, and basophils being associated with atopy to food mix. Furthermore, *D. farinae* was found to be the most prevalent allergen among ATH children, followed by food mix, *D. pteronyssinus*, and *A. alternata*. It suggested that special attention should be paid to ATH children with higher eosinophils, and as the most prevalent allergen, *D. farinae* may be an indicative target of desensitization therapy.

## Data Availability Statement

The raw data supporting the conclusions of this article will be made available by the authors, without undue reservation.

## Ethics Statement

The studies involving human participants were reviewed and approved by this study was approved by the Internal Review Board of the Institutional Ethics Committee of Qilu Hospital of Shandong University (KYLL-2020(KS)-228). Written informed consent to participate in this study was provided by the participants' legal guardian/next of kin.

## Author Contributions

JZ and YW: study design. YY, QF, and HL: data collection. JZ and YY: statistical analysis and manuscript draft. YW and YL: manuscript revised. All authors read and approved the final manuscript.

## Conflict of Interest

The authors declare that the research was conducted in the absence of any commercial or financial relationships that could be construed as a potential conflict of interest.
