# Supplementary material for: Eosinophils Are More Strongly Relevant to Allergic Sensitization Than Basophils in Pediatric Adenotonsillar Hypertrophy
Source: Front Pediatr. 2021 Mar 31;9:598063. doi: 10.3389/fped.2021.598063 (PMC8044536; doi:10.3389/fped.2021.598063)
Supplement: Supplementary file 1 [file Data_Sheet_1.docx]

Table S1. Correlations between levels of sIgE and percentage of eosinophils, basophils, respectively.

|  | Percentage of eosinophils | |  | Percentage of basophils | |
| --- | --- | --- | --- | --- | --- |
|  | r | P |  | r | P |
| *D. pteronyssinus* | 0.262 | <0.001 |  | 0.051 | 0.191 |
| *D. farinae* | 0.248 | <0.001 |  | 0.022 | 0.577 |
| *A. alternata* | 0.322 | <0.001 |  | 0.075 | 0.051 |
| Dander | 0.218 | <0.001 |  | 0.033 | 0.390 |
| Food mix | 0.173 | <0.001 |  | 0.039 | 0.309 |
| Mugwort | 0.185 | <0.001 |  | 0.011 | 0.781 |
| Willow | 0.224 | <0.001 |  | -0.012 | 0.763 |
| Total allergen sIgE score | 0.377 | <0.001 |  | 0.094 | 0.014 |

Table S2. Associations of high percentage of eosinophils with atopy.

|  | Unadjusted | |  | Multivariable adjusted^∗^ | |
| --- | --- | --- | --- | --- | --- |
|  | OR | 95% CI |  | OR | 95% CI |
| *D. pteronyssinus* | 5.480 | (2.535, 11.846) |  | 5.651 | (2.506, 12.743) |
| *D. farinae* | 4.538 | (2.107, 9.776) |  | 4.769 | (2.114, 10.756) |
| *A. alternata* | 2.536 | (1.116, 5.762) |  | 2.592 | (1.115, 6.029) |
| Dander | 1.296 | (0.167, 10.071) |  | 1.224 | (0.154, 9.727) |
| Food mix | 2.150 | (0.949, 4.871) |  | 2.357 | (0.977, 5.689) |
| Mugwort | 5.201 | (1.831, 14.768) |  | 5.287 | (1.794, 15.578) |
| Willow | 6.583 | (2.045, 21.191) |  | 7.700 | (2.271, 26.108) |
| Atopy | 6.734 | (2.528, 17.937) |  | 8.201 | (2.778, 24.206) |
| Polysensitization | 7.340 | (3.069, 17.557) |  | 7.691 | (3.020, 19.585) |

^∗^Adjusted for age, sex, vitamin D, BMI and visiting season.

Table S3. Associations of high percentage of basophils with atopy.

|  | Unadjusted | |  | Multivariable adjusted^∗^ | |
| --- | --- | --- | --- | --- | --- |
|  | OR | 95% CI |  | OR | 95% CI |
| *D. pteronyssinus* | 1.075 | (0.400, 2.890) |  | 1.150 | (0.423, 3.121) |
| *D. farinae* | 0.901 | (0.336, 2.415) |  | 0.940 | (0.347, 2.549) |
| *A. alternata* | 1.410 | (0.558, 3.562) |  | 1.324 | (0.520, 3.368) |
| Dander | 1.920 | (0.350, 10.536) |  | 1.570 | (0.265, 9.323) |
| Food mix | 1.200 | (0.476, 3.024) |  | 1.191 | (0.465, 3.048) |
| Mugwort | 2.657 | (0.757, 9.330) |  | 2.309 | (0.644, 8.282) |
| Willow | 2.692 | (0.593, 12.219) |  | 3.192 | (0.681, 14.957) |
| Atopy | 0.749 | (0.340, 1.647) |  | 0.735 | (0.333, 1.626) |
| Polysensitization | 0.890 | (0.385, 2.054) |  | 0.879 | (0.378, 2.044) |

^∗^Adjusted for age, sex, vitamin D, BMI and visiting season.
